# Peculiar plants and fantastic fungi: An ethnobotanical study of the use of hallucinogenic plants and mushrooms in Slovenia

**DOI:** 10.1371/journal.pone.0245022

**Published:** 2021-01-07

**Authors:** Karsten Fatur

**Affiliations:** Univerza v Ljubljani, Fakulteta za farmacijo, Ljubljana, Slovenia; Ilia State University, GEORGIA

## Abstract

The present study examined the patterns of use among a sample of 68 users of hallucinogenic plants and mushrooms in Slovenia. In compiling the lists of all the participants, 26 different plants/mushrooms, mixtures, or products were found to have been used. The main reason for beginning to use these substances was curiosity, and most people began using them in their 20s. The most used were *Psilocybe* spp., being mentioned by approximately 91% of the participants; 50% of the respondents in the study had made use of no other natural hallucinogens besides these. Many of the plants or mushrooms were used only a small number of times. No matter what items had been used, the internet often played a role in first hearing about them. Dosing and the means of using the various hallucinogens were often quite varied, as were the settings where they were taken. Knowledge of the dangers of these hallucinogenic plants and mushrooms as well as their occurrence in nature were likewise vastly varied. Though public opinion often associates the use of mind-altering substances with problematic drug use and partying, the majority of the individuals interviewed seemed to present a greater desire to experience the interesting effects, to overcome personal difficulties, and for individual and spiritual growth.

## Introduction

Use of hallucinogenic plants and fungi, believed to extend back thousands of years, is an important part of the human experience [1]. Whether used for healing, divination, magic, or protection, these biological wonders have served an important role in the development of cultures and have captured the imagination of many.

Though many words have been used to describe these substances, perhaps the most widely accepted is hallucinogens. The term hallucinogen first became popular in the 1950s and was originally used as the substances were said to produce hallucinations, though we now know that this is often not the case at lower doses [2, 3]. The main effects of these substances are on perception, mood, and thought, and at lower doses they display minimal intellectual impairment and no disabling effects; they are also not physically addictive [4, 5]. These effects are often characterised as being dreamlike and are marked by sensory distortions and sometimes true hallucinations (often visual or audio), exaggeration of emotional state, distortion of time, mystical qualities, and various spiritual experiences and revelations such as ego dissolution, feeling of a near death experience, and a sense of universal connection or understanding [2, 6, 7]. The effect of set and setting (mental state and physical environment) in taking these substances is also known to be important [2].

After a wave of concern in regards to these substances in the 1970s, they came to be used less frequently [7]. They began to witness a revival during the 1990s, one that has been further strengthened by the spread of the internet [8, 9]. As such, their present usage is of great interest from a variety of perspectives, including medicinal, social, legal, and ethnobotanical.

The present study seeks to add to the literature on the modern use of hallucinogenic plants and mushrooms, with a population set in Slovenia. Though the use of mind-altering substances is often broadly stereotyped, the individuals in this study showed a range of motivations for their use, demonstrating that further investigation is warranted from a range of disciplines.

## Methods

Participants were gathered through the dissemination of advertisements both on social media and by posters placed around the capital city of Slovenia. Word of mouth between participants also accounted for many of the recruitments.

Participants were given the option to be interviewed in person or to complete an online questionnaire, for those living further away or uncomfortable sharing the information in person.

Participants were informed of the purpose of the project, how results would be disseminated, and that they could retract their answers prior to publication. They were told that they would remain anonymous, though Dr. Roman Paškulin asked to be named. These steps were undertaken to ensure informed consent, which was obtained verbally in the case of the in-person interviews; for those completing a questionnaire, the information about the project and the informed consent were included before the first questions.

Questionnaires were shared online, and structured interviews based on the questionnaire were used for in-person gathering and were recorded with participant consent. Data was collected between September 2018 and December 2019 and was then analysed qualitatively and with basic statistics.

### Participant information

68 individuals aged 18–60 (mean = 28, SD = 8.43)participated in this study.

52 were male (51 cis-gender, 1 non-binary) and 16 female (all cis-gender). 60 were heterosexual, 3 homosexual, 3 bisexual, one pansexual, and one undefined.

64 were Slovenian, one French/Slovenian, and one Serbian/Slovenian. Two participants were not Slovenian (one Russian and one Serbian), but live within the country.

35 of the participants live in Ljubljana (the capital city) or the surrounding area in central Slovenia. 11 live in the Gorenjska region, 10 in Primorska, 6 in Dolenjska, one in Štajerska, and one in Prekmurje. 2 participants currently reside outside of Slovenia as international students, though they are originally from here.

## Results and discussion

### Age of first use and difficulty to obtain

The average ages of first use for these various plants and mushrooms ranged from 16 to 50, with the average between the substances being 27 (SD 7.39). Data from Europe and America has been stated to consistently show users of hallucinogenic plants to range from 14–56 years of age with a mean of 26, quite close both to the mean age of this sample group [28] and to the average age of first use for all the substances (27) [10].

The youngest first use was *Myristica fragrans* Houtt. (16) and the oldest *Psychotria viridis* Ruiz & Pav (50), though both were listed only once; it may thus be better to use only plants listed by two or more individuals in considering this, thus making the lowest average age of first use *Datura* spp. L. (18) and the oldest *Echinopsis pachanoi* (Britton & Rose) Friedrich & Rowley (35). *Datura* is known to be used by young people in Slovenia and beyond, making this unsurprising, while *E*. *pachanoi* may owe its delayed first use to its foreign home, difficulty to obtain, and high price [9, 11–13].

It is also interesting to note that the average age of first use for local *Psilocybe* (Fr.) P. Kumm. spp. was higher than that for foreign ones; though this may be a result of the smaller sample size for the former group, it could also show that foreign mushrooms served as an introduction to the world of hallucinogenic fungi, with local ones tried later when the individuals were more comfortable with them and felt sufficiently knowledgeable to find and harvest mushrooms themselves.

Participants rated the difficulty of obtaining substances from 1 (easiest) to 5 (hardest). The lowest average ratings were substances collected in nature or bought legally and cheaply; those that were difficult to get came from other countries, unsafe internet sources, shamans or dealers, and often were more expensive. However, the scale was subjective: some rated ayahuasca as 1, while others listed *Psilocybe* spp. as 5. Though this represents each individual's sense, the objective difficulty of obtaining ayahuasca in Slovenia is certainly not lower than that of *Psilocybe* spp.

It often came down to whom the participants knew, with many stating for the more exotic plants that they could be easily obtained with the right contacts. People who used many plants and mushrooms (or used them repeatedly) tended to rate the difficulty of finding such substances lower than those who had used only *Psilocybe spp*. and who had perhaps used them just once. Many individuals also stressed that in the capital it is much easier to find such substances than in rural areas. Many who picked the hallucinogenic plants and mushrooms also noted the importance of the season on availability.

A list of average ages of first use and difficulty to obtain ratings may be seen in Table 1.

**Table 1:** Condensed freelist. * denotes local *Psilocybe* mushrooms.

Name	Listed names	Frequency (/68)	Percent %	Average age of first use	Average difficulty to obtain (1 easiest, 5 hardest)
***Psilocybe* (Fr.) P. Kumm. mushrooms**	*Čarobne gobe*, *Psilocybe semilanceata*, *nore gobe*, magic mushrooms, *zašiljena gologlavka*, *Psilocybe cubensis*, psilocybin, *gobice*, *psilocibin gobice*, *gobe*, *halucinogene gobice*, Psilocybe spp., *čarobne gobice*, *gobe psilocibinke*, Psilocybin mushrooms, *psihadelične gobe*, *čarobne glive*, *Psilocybin gobe*, *Čudežne gobe*, truffles, Psilocybe atlantis forbidden fruit	62	91.18	21 (SD 5.50) [27 {SD 8.50} *]	2 [4*]
***Salvia divinorum* Epling & Játiva**	*Salvija*, Salvia D	16	23.53	22 (SD 8.49)	3
**Ayahuasca**	Ayahuasca	10	14.71	32 (SD 8.96)	3
***Amanita muscaria* (L.) Lam.**	Amanita muscaria, *mušnica*, *Rdeča mušnica*	10	14.71	22 (SD 3.66)	2
***Echinopsis pachanoi* (Britton & Rose) Friedrich & Rowley**	San pedro, Echinopsis pachanoi	7	10.29	35 (SD 10.46)	2
***Datura* L. spp.**	Datura, *Navadni kristavec*, *Datura innoxia*, *Datura stramonium*	6	8.82	18 (SD 2.25)	1
***Ipomoea* L. spp.**	*Ipomoea violacea*, *Ipomoea purpurea*, *Lepi slak*	5	7.35	22 (SD 4.92)	1
***Lophophora williamsii* (Lem. ex Salm-Dyck) J. M. Coult**	Peyote, *pejotl*, *Lophophora williamsii*	5	7.35	27 (SD 13.68)	2
***Tabernanthe iboga* Baill.**	Iboga	3	4.41	33 (SD 15.37)	3
***Argyreia nervosa* (Burm. F.) Bojer**	Hawaiian baby woodrose	3	4.41	20 (SD 4.58)	3
***Atropa belladonna* L.**	*Atropa belladonna*, *volčja češnja*	3	4.41	27 (SD 4.51)	2
***Artemisia* L. spp.**	*Pelin*, *pelinkovec*	3	4.41	21 (SD 7.94)	1
***Peganum harmala* L.**	*Sirijska rutica*, *Peganum harmala*, Syrian rue	3	4.41	27 (SD 7.64)	1
***Scopolia carniolica* (Jacq.) Kuntze**	*Kranjska bunika*	2	2.94	22 (SD 0.71)	1
***Mandragora* L. spp.**	*Nadlišček*, *podlišček*, *Mandragora*	2	2.94	27 (SD 7.07)	3
***Mimosa hostilis* (C. Mart.) Benth.**	*Mimosa hostilis*	2	2.94	27 (SD 4.24)	5
**Changa**	Changa, *Čanga*	2	2.94	29 (SD 4.95)	5
***Psychotria viridis* Ruiz & Pav.**	DMT (*Psychotria viridis*)	1	1.47	50	2
***Spartium junceum* L.**	*Spartium junceum*, *brnistra*, *žuka*	1	1.47	19	1
***Myristica fragrans* Houtt.**	*Muškatni orešček*	1	1.47	16	1
***Hyoscyamus niger* L.**	*Črni zobnik*	1	1.47	22	1
**LSA (powder in a capsule)**	LSA	1	1.47	24	1
***Delosperma acuminatum* L. Bolus**	*Delosperma acuminatum*	1	1.47	37	3
***Phalaris arundinacea* L.**	*Phalaris arundinacea*	1	1.47	38	3
***Laburnum anagyroides* Medik.**	*Nagnoj*	1	1.47	25	/
***Laetiporus sulphureus* (Bull.) Murrill.**	*Žvepleni lepolunknjičar*	1	1.47	28	3

### *Psilocybe* spp.

Perhaps the most well-known natural hallucinogen worldwide, *Psilocybe* mushrooms are members of the Hymenogastraceae and the most common genus of psychoactive fungi [14]. Mushrooms of this genus have a long history of use in shamanic rituals in Mexico and are psychoactive as a result of containing psilocybin, a compound that is then broken down in our digestive system into psilocin, the true hallucinogenic alkaloid [2, 14–16]. Research has consistently shown the use of these mushrooms to be safe with no direct physical damage being caused, though there are low but significant rates of flashbacks and panic attacks associated with taking these fungi, which may make them unsuitable for those with heart issues [14, 16–18]. Research in France with users of hallucinogenic plants and mushrooms showed that all 30 individuals interviewed for a study had previously used *Psilocybe* spp., while in the Czech republic, over 30% of reported toxicology cases were a result of the use of these mushrooms [8, 9].

In this study, *Psilocybe* spp. were the most listed item, appearing 62 times, and had the greatest variety of names. Some were Latin or English; most were Slovene, often including the word for mushrooms (*gobe*, *gobice*) or fungi (*glive*) and adjectives describing their effects (*Čarobne*/magical, *nore*/crazy, *halucinogene*/hallucinogenic, *psihadelične*/psychedelic, *čudežne*/miraculous). Names for all items listed may be seen in Table 1.

Two categories emerged: foreign and local mushroom use. Friends and the internet were the most common ways to first hear of foreign mushrooms, and the main motivation for trying them was curiosity. Use frequency was usually 2–4 times per year. Many noted decline in use as they aged, though whether this is due to a decreased need for exciting experiences or no longer requiring the benefits was often unclear. Continued use was usually due to pleasant and beneficial effects while lack of access and high price were the main reasons for discontinuing. Most ate them whole and dried. The next most common method was grinding dried mushrooms into juice; many believed lemon juice enhanced the trip. Microdoses usually ranged from 0.05–0.2 g, while large doses were usually 3–4 g.

Foreign mushrooms were usually used without other substances, though many combined them with marijuana and occasionally with other intoxicants. They were mainly used in the evening, though there was a preference for daytime use in summer when it was possible to go outside. Participants were roughly divided in half between using them inside or in nature. Some used them at parties. The main condition was usually to be somewhere safe, usually at home or in nature far from populated areas. Weekends were preferred. Most used *Psilocybe* spp. in small groups. Only a few always used them alone.

Foreign mushrooms were usually received from "friends," though one individual noted that dealers often become friends, thus making the exact distinction unclear. Some participants grow foreign mushrooms, usually from grow kits bought online. Most received the mushrooms whole and dried. 5€ per gram and 6–10€ per gram were the most frequently stated prices. Many had difficulty describing the effects; visual alterations involving patterns/shapes/textures were most reported. Dangers were mainly mentioned in relation to predispositions to mental health issues, especially schizophrenia, followed by the danger of having a bad trip, though this effect was infrequently reported in the present study. Some believed that these exact mushrooms grew in Slovenia, clearly mistaking them for local varieties.

Five participants used local *Psilocybe* spp. (likely *P*. *semilanceata* [Fr.] P. Kumm. based on descriptions, though it is worth noting that nearby countries have published ecological findings that suggest a richness in the variety of hallucinogenic fungal species in the area [19]), with one never having used foreign ones. Dosing ranged from 3–25 mushrooms, or 1–5 g, significantly lower than in previously published research from Poland [18]. All but one participant picked the mushrooms themselves. They were often compared to foreign mushrooms but said to be stronger. Participants stated that they are not hard to get, but you need to know when and where they grow and how to identify them, as well as having a means of getting to them.

2 participants who used foreign *Psilocybe* spp. also listed *Psilocybe* truffles. Both got them in Amsterdam or from someone who had been there and described them like foreign *Psilocybe* spp.

With all *Psilocybe* mushrooms together, they were one of the few plants or mushrooms to be the sole element listed by some. These were the most often to occur in this sense, being the sole item listed by 34 individuals. They were co-listed with *Salvia divinorum* Epling & Játiva 16 times, the highest frequency of co-occurrence between any listed items. The complete analysis of co-listing for all plants and mushrooms may be seen in Table 2.

**Table 2:** Freelisting co-occurrence table. Represents the number of times two given plants were found on a participant's list together.

	Listed alone	*Psilocybe* spp.	*Salvia divinorum*	Ayahuasca	*Amanita muscaria*	*Datura* spp.	*Echinopsis pachanoi*	*Ipomoea* spp.	*Lophophora williamsii*	*Tabernanthe iboga*	*Argyreia nervosa*	*Atropa belladonna*	*Artemisia* spp.	*Peganum harmala*	*Scopolia carniolica*	*Mandragora* spp.	*Mimosa hostilis*	Changa	*Psychotria viridis*	*Spartium junceum*	*Myristica fragrans*	*Hyoscyamus niger*	LSA powder in capsule	*Delospermum acuminatum*	*Phalaris arundinacea*	*Laburnum anagryoides*	*Laetiporus sulphureus*
*Psilocybe* spp.	34		16	8	9	5	6	4	5	3	3	3	1	3	2	2	2	2	1	1	1	1	1	1	1	1	1
*Salvia divinorum*	0	16		3	6	4	4	3	2	2	2	2	1	0	0	0	2	1	1	0	1	0	0	1	1	1	1
Ayahuasca	1	8	3		3	1	5	0	2	2	1	1	0	1	0	1	1	0	1	0	0	0	0	0	0	0	0
*Amanita muscaria*	0	9	6	3		4	4	0	3	2	1	3	1	0	1	1	1	1	1	0	0	1	0	1	1	1	0
*Datura* spp.	0	5	4	1	4		3	1	3	1	0	2	0	0	1	1	1	0	1	0	0	1	0	1	1	1	0
*Echinopsis pachanoi*	0	6	4	5	4	3		0	3	1	1	1	0	0	1	1	1	0	1	0	0	1	0	1	1	0	0
*Ipomoea* spp.	0	4	3	0	0	1	0		0	0	1	0	1	0	0	0	0	0	0	0	0	0	0	0	0	0	0
*Lophophora williamsii*	0	5	2	2	3	3	3	0		2	0	1	0	1	1	2	0	0	1	0	0	1	0	1	1	0	0
*Tabernanthe iboga*	0	3	2	2	2	1	1	0	2		0	0	0	1	0	1	0	0	1	0	0	0	0	0	0	0	0
*Argyreia nervosa*	0	3	2	1	1	0	1	1	0	0		0	1	1	0	0	1	0	0	1	0	0	0	0	0	0	0
*Atropa belladonna*	0	3	2	1	3	2	1	0	1	0	0		0	0	1	1	0	0	0	0	0	1	0	0	0	1	0
*Artemisia* spp.	1	1	1	0	1	0	0	1	0	0	1	0		0	0	0	0	0	0	0	0	0	0	0	0	0	0
*Peganum harmala*	0	3	0	1	0	0	0	0	1	1	1	0	0		0	1	0	0	0	1	0	0	0	0	0	0	0
*Scopolia carniolica*	0	2	0	0	1	1	1	0	1	0	0	1	0	0		1	0	0	0	0	0	1	0	0	0	0	1
*Mandragora* spp.	0	2	0	1	1	1	1	0	2	1	0	1	0	1	1		0	0	0	0	0	1	0	0	0	0	0
*Mimosa hostilis*	0	2	2	1	1	1	1	0	0	0	1	0	0	0	0	0		0	0	0	0	0	0	0	0	0	0
Changa	0	2	1	0	1	0	0	0	0	0	0	0	0	0	0	0	0		0	0	0	0	0	0	0	0	0
*Psychotria viridis*	0	1	1	1	1	1	1	0	1	1	0	0	0	0	0	0	0	0		0	0	0	0	0	0	0	0
*Spartium junceum*	0	1	0	0	0	0	0	0	0	0	1	0	0	1	0	0	0	0	0		0	0	0	0	0	0	0
*Myristica fragrans*	0	1	1	0	0	0	0	0	0	0	0	0	0	0	0	0	0	0	0	0		0	0	0	0	0	0
*Hyoscyamus niger*	0	1	0	0	1	1	1	0	1	0	0	1	0	0	1	1	0	0	0	0	0		0	0	0	0	0
LSA powder in capsule	0	1	0	0	0	0	0	0	0	0	0	0	0	0	0	0	0	0	0	0	0	0		0	0	0	0
*Delospermum acuminatum*	0	1	1	0	1	1	1	0	1	0	0	0	0	0	0	0	0	0	0	0	0	0	0		1	0	0
*Phalaris arundinacea*	0	1	1	0	1	1	1	0	1	0	0	0	0	0	0	0	0	0	0	0	0	0	0	1		0	0
*Laburnum anagryoides*	0	1	1	0	1	1	0	0	0	0	0	1	0	0	0	0	0	0	0	0	0	0	0	0	0		0
*Laetiporus sulphureus*	0	1	1	0	0	0	0	0	0	0	0	0	0	0	0	0	0	0	0	0	0	0	0	0	0	0	

Responses are summarised in Table 3.

**Table 3:** Overview of *Psilocybe* spp.

	Foreign *Psilocybe* spp.	Local *Psilocybe* spp.
How first heard of	Friends [37], internet [20], common knowledge [7], parents [4], school [4], movies [4], books [3], siblings [2],	Internet [2], friends [2], common knowledge [1], books [1]
Why first tried	Curiosity [25], fun [4], experimenting [4], self-exploration [4], access another reality [3], because of friends [3], diminish ego [2], self-improvement [2], wanting to try something new [1], starting point in hallucinogen use [1], spiritual experience [1], spiritual growth [1], transcendental experience [1], compare to LSD [1], heard of good experiences of others [1], explore altered states of mind [1], boredom [1], understand hype about mushrooms [1], to cure depression/anxiety [1], alternative to ecstasy [1], alternative to alcohol [1], alternative to marijuana [1]	Curiosity [3], happened to have it around [1], wanted to experiment with a safe hallucinogen [1]
Number of times used	2–4 times per year [15], once per year [13], twice [9], once [4], 3 times [3], once per month [2], 1–2 times per year plus microdoses when feeling depressed [2], 4–6 times per year [2], when the urge comes [2], microdosing for a month [1], once every 2–3 years [1], 5 times [1], microdosing for 3 months [1], 2–3 times per month [1], 4 times [1]	1–2 times per year [2], once [1], 3 times [1], 5–6 times [1]
Reasons for discontinuing use	Lack of access [4], price [2], knowing someone who got arrested for having them [1], preferring LSD [1], effects not desirable [1], microdosing didn't help depression [1], microdosing didn't have desired effects [1], started using local *Psilocybe* spp. instead [1]	Availability [1]
Reasons for continuing use	Pleasant effects [16], beneficial effects [14], trying to recreate first experience [4], wanting to discover more [3], seeking new perspectives [3], experiment with different doses [3], fun [3], spiritual benefits [2], ability to connect users to earth/nature [2], helping with depression symptoms [2], took too little the first time [2], reset from daily life [1], seeking enlightenment [1], repeat experience in different surroundings [1], due to the short trip [1]	Stronger than foreign *Psilocybe* spp. [2], prefers effect to foreign *Psilocybe* spp. [2], improves mental state [1], to help with depression [1]
Method of consumption	Eating whole dry mushrooms [43], dry mushrooms ground in juice [15], dry mushrooms in tea [8], dry mushrooms powdered in capsules [7], dry mushrooms eaten with food [4], dry mushrooms ground into water [3], dry mushrooms made into smoothie [2], dry mushrooms eaten with honey [2], mixed with alcohol [2], eating fresh mushrooms [1], smoking dried mushrooms [1], dry mushrooms ground into milk [1]	Eating whole dry mushrooms [3], whole dry mushrooms in tea [1], whole dry mushrooms ground into juice [1]
Dose	Microdose: 0.05–0.2 g [6], 0.3–0.6 g [2], half a mushroom head [1].	Microdose: single mushroom [1].
	Full doses: 3–3.9 g [14], 1–1.9 g [12], 2–2.9 g [12], 4–4.9 g [7], 5–5.9 g [3], 8 g [3], 13 g [1], less than 1 g [1], 10 small mushrooms [1], 2 big mushrooms [1], walnut-sized volume [1]	Full dose: 3 mushrooms [1], 20–25 mushrooms [1], 1 g [1], 2–5 g [1]
Use with other substances	Usually alone [42], usually with marijuana [13], occasionally with marijuana [10], occasionally with alcohol [7], occasionally with marijuana during the come down [4], with MDMA a couple times [2], with *Peganum harmala* tea sometimes [2], with changa once [1], with amphetamine occasionally [1], with LSD once [1]	Alone [4], once with LSD [1]
Time of use	Microdoses: morning [3]	Afternoon [3], day [1], no pattern [1]
	Full doses: Evening [25], day [14], weekend [12], warmer months [10], whenever convenient [7], morning [6], time not important [4], when the urge strikes [3], based on lunar/astrological factors [1].	
Place of use	Nature [35], inside [29], parties [5]	At home only [2], inside and outside [2], only outside [1]
Use with others or alone	With others [38], sometimes alone and sometimes with others [14], alone [6]	Alone [2], with others [2], alone and with others [1]
Methods of obtaining	Friends [38], grow them at home [11], dealer [4], buy online [4], picked in Brazil [1], bought in Amsterdam [1]	Picked them [4], from friends [1]
State when obtained	Dry and whole [47], dried in pieces [9], grow box/mycelium [7], powdered [3], capsules [2], fresh [2], picked fresh [1]	Fresh [4], dried [1]
Cost	5€/g [22], 6–10€/g [22], free [7], 40–70€ for a grow kit [5], 11–15€/g [2], a couple euros [1]	Free [5]
Effects	Visual alterations of pattern/shape/texture [29], nausea [16], seeing intense colours [14], revelations/contemplation about self [15], altered way of seeing/looking at things [15], sense of everything in the environment flowing/melting [11], laughing [11] increased thinking [9], euphoria [8], hyperawareness/noticing things not usually noticed [8], stretching of time [7], sense of connection to universe [7], altered thinking [7], changed colours [6], closed eye visuals [6], ego death/weakening [6], warmth in body [5], fatigue [5], realistic hallucinations [4], sense of softness/bendiness to body [4], enhanced mood [4], feeling of being in another world [4], connection to nature [4], heaviness of body [4], distorted sounds [4], sense of connection to others [4], increased emotionality [3], light-headedness [3], increased imagination [3], confusion [3], fear [3], hyper focus [3], enhanced creativity [3], seeing sounds [3], inability to concentrate [3], sweating [3], enhanced sense of hearing [3], stretching of space [2], difficulty moving [2], difficulty breathing [2], fidgety [2], faces shifting [2], inability to distinguish past from present [2], out of body experience [2], feeling of everything being ok [2], increased empathy [2], sense of purpose/meaning [2], mystical feeling [2], disorganised thinking [2], problems seeming less serious [2],seeing auras/energy [2], changed awareness of self [2], feeling of flying/floating [2], weakness [2], laziness [2], telepathy [2], jitteriness [2], sense of body expanding [1], heightened sense of touch [1], disorientation [1], time seeming to move faster [1], sense of body disintegrating [1], sense of relationships being restructured [1], feeling refreshed [1], panic [1], flashbacks to previous trips [1], loss of sense of boundary between self and environment [1], religious motifs and thoughts [1], fear of never returning to normal [1], increased energy [1], vomiting [1], increased strength [1], sense of space shrinking [1], bad trip [1], communicating with entities [1], intense internal monologue [1], decreased stress [1], fantasy state [1], feeling of going crazy [1], difficulty sleeping [1], feeling of being able to see through things [1], sense of inner peace [1], enhanced sense of smell [1], difficulty separating thoughts from reality [1], decreased motor skills [1], difficulty walking, sense of mind being shattered [1], enthusiasm [1], dissociation [1], trouble speaking [1], feeling of having super powers [1], decreased fatigue [1], feeling of wet hands and feet [1], feeling of self-sufficiency [1], deterred from consumerism [1], playful/child-like feelings [1], tightness in body [1], feeling animalistic [1], calmness [1], anxiety [1], spiritual feeling [1], increased understanding of others [1], inner guiding voice [1], tingling in fingers [1], clearer thinking [1], lost in thought [1], sense of equality of all things [1], music playing in head [1], higher understanding [1], self-critical [1], magical experience [1], getting stuck in memories [1]	Hyper-real vision [1], euphoria [1], more hallucinations than foreign *Psilocybe* spp. [1], increased emotionality [1], difficulty concentrating [1], confusion [1], more nausea than foreign *Psilocybe* spp. [1], auditory effects [1], same effects as foreign *Psilocybe* spp. but stronger [1]
Dangers	Dangerous to those with mental health issue or predisposition [17], bad trips [14], if not in the right mental space [10], if taken in a bad environment/bad company [10], if you take too much [10], can be physically hurt while tripping [8], if you don't know what you are getting into [4], if not used safely [4], nausea/vomiting/diarrhoea [3], if used when alone [3], none [2], paranoia [2], panic [1], if taken by accident [1], if from an unknown source [1], derealisation [1], may react with some medications [1], if you eat them when mouldy [1]	Can be physically hurt while tripping [2], none [2], misidentification when picking [1]
Natural knowledge	Related species grows in Slovenia [22], various related species grow worldwide [10], they grow in Slovenia [10], grow on manure [5], grow in cow pastures [3], from Mexico [2], from South/Central America [2], grow at high altitudes [1], from Southeast Asia [1], from India [1], from tropical climates [1], from Africa [1], 12–15 related species grow in Slovenia [1], from Scandinavia [1], from Siberia [1], from Europe [1]	Small mushrooms growing in cow pastures on manure that have a darkened "nipple" on the top [5]

### Salvia divinorum

A rare member of the Lamiaceae that grows in only a portion of Oaxaca, Mexico, this plant has long been used for medicinal preparations and as a visionary plant in divination and shamanic training [20–23]. Its psychoactivity results from salvinorin-a, a k-opioid receptor agonist that is thought to be the most powerful natural hallucinogen [21, 24–26]. Research shows that the most common means of using this plant recreationally involves smoking the dried leaves or extracts, and that this produces a short (1–20 minute) but intense trip [23, 26–29]. The effects are characterised by audio and visual hallucinations as well as a strong sense of dissociation from reality and/or the self and senses of the body changing and/or merging with elements of the environment; overall it is described as an unmatchable experience [23, 26–30]. However, many individuals report no effects their first time, with subsequent attempts necessary before they are obtained [31]. Research has shown that most individuals using *S*. *divinorum* are young (aged 22 or less) and usually male [28, 30]. It is often used once or twice, with low availability cited as a reason for discontinuing use [8].

In the present study, *Salvia divinorum* was the second most used substance, listed 16 times. It was mainly first heard of on the internet. Curiosity was the main reason to try it. Most used this plant once before discontinuing due to availability or lack of desirable effects. 2 chewed the leaves, one made and smoked an extract from leaves, and all other participants smoked dried leaves. Smoking was often communal and thus the exact amount ingested unknown. Most used this plant without other substances; a couple included tobacco to their smoking mix, while alcohol, marijuana, and MDMA were also sometimes combined. The plant was almost always used with other people, mainly outside.

*Salvia divinorum* was most frequently obtained as whole, dried leaves, though plant cuttings were also often mentioned. Most participants received the plant free from friends. One participated in a ritual involving this plant and that cost between 100 and 250€. Buying this plant on the internet was said to be risky.

Effects were described as lasting about 5 minutes and being intense when smoked, or lasting a couple of hours and being mild when chewed. An intense high was the most reported effect along with euphoria, though directly following this were claims that there had been no effect. The most listed danger of this plant was causing harm to yourself while high.

Responses are summarised in Table 4.

**Table 4:** Overview of *Salvia divinorum* and ayahuasca.

	*Salvia divinorum*	Ayahuasca
How first heard of	Internet [7], YouTube [3], friends [6], common knowledge [1], books [1], "ganja shop" [1]	Books [4], internet [4], friends [2], told by travellers they met in Peru [1], told by someone who knew a shaman in Slovenia [1]
Why first tried	Curiosity [9], offered it at a party [2], searching for a substitute for marijuana [2], growing it at home and not wanting to waste the leaves [1], experience another reality [1], friend bought too much [1]	Curiosity [2], self-discovery [2], invited to try it [1], spiritual purposes [1], experience another reality [1], diminish ego [1], to cure depression [1], self-improvement [1], felt it was calling to them [1]
Number of times used	Once [9], twice [4], multiple times [3]	Once [2], 5–7 times [2], once or twice per year [3], twice [1], once per month [1]
Reasons for discontinuing use	Availability [4], no effects [2], weak effects [2], scared of the plant after trying it [1], no effects first time and uninteresting effects second time [3], severe headaches [1], too aggressive on the body [1], no longer felt the need to get high [1], smoking the leaves is disrespectful to the gods of the plant but chewing them is too much work [1]	Expensive [2], draining/long experience [2], starting a meditation practices that forbids mind-altering substances [1], difficult to find in Slovenia [1], got the answers they wanted [1]
Reasons for continuing use	No/weak effects first time [7]	Desirable effects [2], spiritual and healing effects [1], to connect with the universal consciousness [1], gives clear idea of life direction [1], need to use a few times to get the healing effects [1]
Method of consumption	Smoking dried leaves [13], chewing leaves [2], smoking homemade leaf extract [1]	Mixed beverage [3], beverage from the leaves of *Psychotria viridis* and the bark of *Banisteriopsis caapi* [2], ayahuasca root made into a beverage [1], beverage from two plants to include MAOI and DMT though shaman may add in other plants as well [1], beverage that uses various plants such as *Mimosa hostilis* root bark [1], brew or a tea made from just the root bark of a liana or this root bark and chacruna leaves [1]
Dose	8 leaves chewed [2], 1 g dried leaves [2], one bong bowl [2], one joint shared among many people [1], several leaves [1], coin-sized amount of dried and crushed leaves [1], one dried leaf [1], a pinch [1], 10 g of leaves made into 1 g of extract [1], 1 g of dried leaves in a joint split between people [1], small amount [1]	As the shaman says [3], depends on the strength [2], 3 cm in bottom of plastic cup [1], 50 ml [1], 100–120 ml or 40 ml if repeating use in the same year [1], 1 cup [1], at own discretion [1], 1–2 teaspoons [1], 300–600 ml [1]
Use with other substances	Alone [10], tobacco [2], alcohol [2], marijuana after effect wore off [1], when high on MDMA [1]	Alone [7], sometimes with hapé [1], once with *Echinopsis pachanoi* [1], once with *Erthroxylum* leaves [1]
Time of use	Evening [5], day [2], night [1], morning [1], weekend [1], spring/summer [3]	Evening into night [2], night [2], evening [1], summer [1], usually daytime [1], weekends [3]
Place of use	Outside [6], home [4], friend's house [2], parties [2], cinema [1], ritual [1]	Workshops/ceremonies in Slovenia [6], ceremony in Peru [3], in nature in Slovenia [2], ceremony in Brazil [1], cottage in Slovenia [1], home [1]
Use with others or alone	Groups [8], alone [5], with a trip sitter [1]	With others [7], alone [1], alone in Slovenia or with others in Peru [1]
Methods of obtaining	Friend [9], online [2], shaman [1], "ganja shop" [1], cannabis shop [1], friend of father [1]	Workshop/ceremony in Slovenia [6], ceremony in Peru [2], shaman in Slovenia [1], shaman in South America [1], ceremony in Portugal [1], shaman in Sweden [1], ceremony in Brazil [1], shaman in Brazil [1], from a friend who brought it from Colombia [1], bought in Slovenia [1]
State when obtained	Whole dry leaves [7], cuttings [6], dry crushed leaves [2]	Prepared beverage [9]
Cost	Free [10], 5€/g [1], 5€/cutting [1], 100–250€ for a ritual with it [1]	100€ for a workshop/ceremony in Slovenia [2], 300€ for a workshop/ceremony in Slovenia [2], 40–50€ in Slovenia [1], 100€ in Europe [1], 400–500€ for an ayahuasca retreat in Slovenia [1], 100–250€ for a ceremony [1], 300€ for a weekend retreat with two doses [1], 200 USD to buy 1 L in Peru [1], 150€ for a ceremony
Effects	Intense high [3], euphoria [3], smiling and laughing [2], no effect [2], feeling of melting into environment [2], changed thinking [2], gross bitter taste when chewed [1], no effects when chewed [1], similar to marijuana but weaker [1], laziness [1], altered sense of balance [1], subtle shift in vision [1], altered perception [1], inability to move [1], intense visual distortion [1], enhanced mood for hours after [1], relaxation [1], loss of sense of place [1], loss of sense of time [1], zoning out [1], dream-like visuals [1], heavy body [1], light-headedness [1], confusion [1], heavy head [1], subtle spooky feeling [1], difficulty concentrating [1]	Vomiting [4], experience different every time [3], sense of oneness with nature [3], extreme nausea [3], more introspective than mushrooms [2], seeing sounds [2], see entities [2], sense of body disintegrating [2], difficult to move/gravity feels stronger [2], all-or-nothing trip [1], relive life [1], feeling of support/love from nature [1], ego dissolution [1], feeling of being put together differently after [1], feeling that whole life led to the moment of taking ayahuasca [1], understanding of universe [1], bliss [1], uncomfortable feelings [1], heaviness in stomach [1], most intense experience in life [1], extreme hallucinations [1], sense of being observed by a greater being [1], feeling of being in the womb [1], seeing hidden fears [1], visual and auditory hallucinations [1], fractals [1], sense of travelling through the universe [1], similar to other sources of DMT but with longer effects [1], diarrhoea [1], introspection [1], closed eye visuals [1], weak open eye distortions [1], disorientation [1], distortion of time [1], filters on perception removed [1], meet spirits of plants/animals/dead/ancestors [1], senses more alert [1], see overlapping realities [1], see personal past as if watching a movie [1], see personal past from view of an older self or one's parents [1], understand in new way [1], telepathy [1], hearing light [1], feeling of having more than 5 senses [1]
Dangers	Losing touch with reality and getting hurt [5], paranoia [2], none [2], psychologically dangerous [2], panicking and getting hurt [1], may trigger schizophrenia [1], dangerous [1]	Bad trips [2], strict diet avoiding MAOIs must be observed [2], none [1], for people with mental health issues [1], for people taking psychiatric medications [1], if combined with recreational drugs [1], people sometimes wander off and disappear when high [1], vomiting/diarrhoea [1], for people with heart problems as it is a very intense experience [1]
Natural knowledge	From South America [4], most plants around the world clones of just 2–3 plants [3], looks like other mint family plants [2], looks like sage [2], rarely flowers [2], from a small region in Mexico [1], reproduces when stems fall and root [1], rarely produces seeds [1], related to sage that one uses when sick [1], from Central/South America [1], evolved closely with humans and not really found in nature [1], from Mazatec region in Mesoamerica [1], Rare plant [1], grows in jungles [1], has long oval leaves [1], from Central America [1], grows in Latin America and Asia and possibly Europe [1]	From South America [2], plants used grow in the jungle [2], 2 plants mixed together [1], one plant contains DMT and another something that stops the DMT from being broken down [1], also called yagé [1], different names in different places [1], chacruna one of the plants in it [1], mixture of the bark of a vine and leaves of another plant [1], combination of *Banisteriopsis* and *Psychotria* [1], made from a vine that sometimes grows as a bush [1], vine from the Amazon [1]

### Ayahuasca

Unlike the majority of items here discussed, ayahuasca is not a plant, but rather a mixture. Native to the Amazon, ayahuasca is a beverage traditionally used by shamans to help with divination and healing; there is a large ritual system built around using this mixture, which serves an important social-cohesion function [32, 33]. Though the ingredients used to create ayahuasca vary, the generally mentioned main ingredients are the bark of the vine *Banisteriopsis caapi* (Spruce ex Griseb.) Morton and the leaves of *Psychotria viridis* [5, 34]. *P*. *viridis* leaves are a source of N,N-dimethyltryptamine, better known as DMT; this substance works on serotonin receptors to cause its effects [35, 36]. However, it is not orally active as it is decomposed in the stomach; for it to be effective when ingested orally, it must be taken with a monoamine oxidase inhibitor (MAOI) that prevents rapid breakdown [16, 36]. *B*. *caapi* bark is rich in harmala alkaloids such as harmine and harmaline, which function in exactly this manner [35, 36]. Combining the two creates a beverage that is orally active as a hallucinogen. As these two plants grow in a limited range of the world's tropical habitat, many have created "ayahuasca analogues" by combining other more widespread plants that contain the same alkaloids [37].

Ayahuasca became popular outside the Amazon in the 1990s, and since has seen a rise in use around the world, as well as in "ayahuasca tourism," in which individuals travel to the Amazon in order to participate in rituals [34, 37]. Though ayahuasca tourism is criticised as being a radicalised form of recreational drug use, research suggests that users undergoing these voyages do so seeking spiritual growth or to help with psychological conditions [38, 39].Though legally questionable in many countries outside of Amazonia, its status as a spiritual sacrament for some recognised religious groups has led to increased acceptance [40–46]. Previous research in France has suggested that usage of ayahuasca is low, with only 4 out of 30 individuals in a study having made use of it [8].

10 participants used ayahuasca in the present study. Books and the internet were the main means of hearing about it, while curiosity and self-discovery were the main reasons for trying. Use frequency varied immensely. High price and the difficulty of the experience were the main reasons to cease use; increased spirituality and personal healing were cited as reasons for regular use.

Dosage varied, and one participant noted that less is needed as you become experienced in using it. Most used ayahuasca without other substances, though hapé (a psychoactive snuff), *Echinopsis pachanoi*, and *Erythroxylum* P. Browne leaves were sometimes consumed with it, as directed by shamans. Most said ayahuasca should be taken in the evening so the effect runs through the night and that there should be free days after taking it. Most used ayahuasca in the presence of others.

All received ayahuasca as a prepared beverage, buying it directly or participating in ceremonies in Slovenia and abroad. Workshops/rituals in Slovenia were mainly suggested to cost 100–250€. Buying ayahuasca independently in Slovenia was said to cost between 40–100€. Participants stated that it is easier to find the past few years in Slovenia, and that it is not difficult once you have connections. The price of rituals was listed as a barrier.

The most listed effect of ayahuasca was vomiting. One participant felt ayahuasca was completely safe, but the others listed various dangers.

Ayahuasca was one of the few substances to be listed alone, with one person who had used it never having tried other hallucinogenic substances.

Responses are summarised in Table 4.

### Amanita muscaria

This Agaricaceae mushroom was traditionally used as a ritual hallucinogen in Siberia [35, 47]. Its psychoactivity is caused by ibotenic acid and muscimol [48]. Muscimol is more hallucinogenic, and seasonal variation in the strength of this mushroom likely results from shifting ratios between these substances [49]. Effects are dependent on preparation, as dehydration changes ibotenic acid into muscimol, which is also more readily extracted in water; water infusions may help leave behind substances responsible for unpleasant physical effects [48]. Previous research from France and the Czech republic have shown minimal use, though it has been becoming increasingly popular in Poland over the recent years [8, 9, 50].

In the present study, 10 participants listed *Amanita muscaria* (L.) Lam. The internet was the main introduction to this mushroom as a hallucinogen. Most heard of it from family members as children and believed it to be poisonous. Curiosity was the main reason to try. Only one participant uses the mushroom regularly, the others having discontinued due to nausea. Given the importance of preparation, it is unsurprising these individuals had undesirable experience–only one individual dried the mushroom and then infused it into water, while all others directly consumed it. Most noted the taste to be disgusting. Dosing ranged from an 8 cm diameter portion of the cap up to 1.5 mushrooms. It was mainly used without other substances, with one participant even noting that it absolutely must be taken alone and cautioned that the mushrooms could be greatly varied in strength. It was almost exclusively used in nature while in the presence of others.

The most frequently listed effect was nausea. Multiple participants stated that their perception shifted in significant ways, but without actual hallucinations. Half suggested it could be dangerous in ways related to the nausea it causes. Most described the mushroom as red with white spots, stating that they grow in forests. The northern region of Slovenia was said to be a good area for finding them.

Responses are summarised in Table 5.

**Table 5:** Overview of *Amanita muscaria* and *Echinopsis pachanoi*.

	*Amanita muscaria*	*Echinopsis Pachanoi*
How first heard of	Internet [3], Erowid [1], books [1], friends [1], folk tradition [1]	Books [2], internet [2], local shaman [1], shaman in Peru [1]
Why first tried	Curiosity [4], mushroom research [1], wishing to join into the folk tradition [1],	Curiosity [3], experience another reality [1], diminish ego [1], personal growth [1], asked by a friend to do it with them [1], to feel the power of the plant [1]
Number of times used	Once [3], 3 times [2], multiple times [1]	Once [3], 2–3 times [2], about twice per year [1]
Reasons for discontinuing use	Disliked effect [1], easier to use *Psilocybe* spp. [1], not interesting experience [2]	Nausea [1], only tried it as part of an ayahuasca ceremony [1], availability [1], disliked effects [1], didn't feel the need for another experience [1]
Reasons for continuing use	Lack of effect so dose was upped [2], the power of the mushroom [1]	Weak effect on first try [1]
Method of consumption	Eat whole dry mushrooms [2], eat whole fresh mushroom [1], eat fresh caps [1], caps dried and made into tea [1], cap dried after skin was removed and then powdered into water and drank [1]	Homemade extract [1], dried skin of cactus and then made into drink [1], made into smoothie [1], cooked and eaten with lemon [1], tea [1]
Dose	10 cm diameter piece of cap [1], 8 cm diameter piece of cap [1], 1.5 mushrooms [1], 1 big mushroom [1]	0.5 g dried plant [2], 4 cm of liquid in the bottom of a cup [1], 100 ml of beverage [1], 200 ml of beverage [1]
Use with other substances	Alone [5], while hungover from alcohol and high on marijuana [1]	Alone [5], with ayahuasca [1]
Time of use	Variable [2], morning [1], afternoon [1], evening [1], weekend [1]	Evening [2], night [1], afternoon [1], summer [1]
Place of use	Outside [4], inside [2]	At home [3], outside [2], inside during ceremony [1]
Use with others or alone	With others [5], alone [1]	With others [4], alone [1]
Methods of obtaining	Collected in nature [7]	Shaman [3], internet [2], acquaintance [1], grown at home [1]
State when obtained	Fresh [7]	Beverage [3], fresh [2], dried and cut [1]
Cost	Free [7]	Unsure [3], 100–250€ per ceremony, 100–200€/kg [1], 8€ per plant [1]
Effects	Nausea [3], no stable set of hallucinations [1], decreased thinking [1], strange feeling in mouth [1], warmth in body [1], increased energy [1], intense sensory effects [1], tingling of hands [1], confusion [1], euphoria [1], confused perception [1]	Nausea [2], positive feelings [1], similar to LSD [1], visual distortion [1], cognitive distortion [1], meeting the male soul of the plant [1], fatigue [1], dream-like state [1], similar to mushrooms [1], dissolving of ego [1], red tinge to vision [1], no obvious peak to high [1], like ayahuasca [1], slight distortion of reality [1], sense of connection to world [1], antidepressant after effects [1]
Dangers	If too much is taken [2], hard on the stomach [1], if used foolishly [1], hard on the kidneys [1], vomiting [1]	None [1], unsure [1], dangerous [1], probably dangerous [1], psychological danger [1]
Natural knowledge	Red cap with white dots [3], grows in forests [2], grows in Slovenia [2], grows around pine [1], grows around fir [1], grows in the forests in northern Slovenia [1], does not grow around beech [1], forms symbiotic relationship with trees [1]	Cactus [1], from tropical climates [1], requires abundant sunlight [1], can grow several metres tall [1], cross section usually a 5-pointed star [1], from Peru [1]

### Echinopsis pachanoi

This member of the Cactaceae is made hallucinogenic by mescaline, a phenethylamine [14]. Research in France has shown that less than a third of hallucinogenic plant users had tried this plant, with those who had done so electing to consume it as a tea or by cooking the cactus to make a sort of dough that was swallowed [8].

7 participants used this plant in the present study. Books and the internet were the main ways of finding out about this cactus; the main motivation for trying it was curiosity. Three used this plant only once, mainly discontinuing due to availability. Those who took it 2–3 times were seeking a better effect than the first trip, but stopped due to unpleasant effects such as nausea, the most commonly listed effect.

Mainly consumed as a beverage, dosing was described various ways. One participant took this plant during an ayahuasca ceremony, but the others used it without other substances; one individual stressed that it must be taken alone. All but one participant used the cactus in the presence of other people and use was primarily inside.

Most participants obtained *E*. *pachanoi* from a shaman, though one grows it at home after buying it in a local shop where it was being sold as a houseplant. Participants were divided on whether or not the cactus is dangerous.

Responses are summarised in Table 5.

### *Datura* spp.

As with the other plants from the family Solanaceae in this study, *Datura* spp. are hallucinogenic and highly toxic as a result of the anticholinergic tropane alkaloids hyoscyamine and scopolamine, which act as muscarine receptor antagonists [36, 51]. These plants have a long history of recreational use; in parts of France, the seeds were mixed with cider for farmers to consume after a day's work [52]. More recent work in France has found that almost half of hallucinogenic plant users have experimented with *Datura*, with many of them using it only once due to the "dark" and negative experiences [8]. Toxicology centres in France have likewise seen many cases as a result of the abuse of these plants, a trend also seen in other countries such as Spain, Germany, Poland, and the Czech Republic [9, 10, 53–55]. Toxicology reports in Slovenia have had similar findings, with *D*. *stramonium* L. being the highest source of drug-related plant intoxications in the country [11]. These sources point toward one-time usage among teens, often as a result of peer pressure, as being the main source of drug use for *Datura* spp.

In the present study, 6 individuals used *Datura* spp. All used *D*. *stramonium*, while one also used *D*. *innoxia* Mill., which they stated was stronger. Half used it only once, with bad experiences causing discontinuation. One participant used repeated microdoses to enhance their dreaming but discontinued after an accidental large dose. Only one participant uses the plant regularly, doing so twice a year to obtain a state of inner peace.

Many learned of *Datura* spp. by knowing people who had taken it and curiosity was why many tried it. Seeds were usually used, generally 2–10. Only one participant took it while alone. One individual grew the plant, while the others collected it in nature. Slovenia's coastal region was noted as a place where it grows abundantly, though I have personally observed it far more often in the central region of the country.

The most commonly listed effects were loss of touch with reality and temporal distortion. Many other negative effects were listed; one participant wandered naked through the city, waking 40 km from home. Participants agreed that the plant is dangerous, with one specifying that the recreational dose is close to the toxic dose.

Responses are summarised in Table 6.

**Table 6:** Overview of *Datura* spp. and *Ipomoea* spp.

	*Datura* spp.	*Ipomoea* spp.
How first heard of	From others who had used it [3], books [1], Erowid [1], folk tradition [1]	Internet [3], Erowid [1]
Why first tried	Curiosity [3], lack of access to other substances [1], dream research [1], desire to join folk tradition [1], peer pressure [1]	Curiosity [2], interest in effects [1], wanting to compare to LSD [1], seeking religious experience [1]
Number of times used	Once [3], twice [1], series of microdoses and one larger dose [1], twice per year [1]	3 times [2], once [1], 5–6 times [1]
Reasons for discontinuing use	Bad experience [5]	Effects not desirable [2], fear of bad trips [1]
Reasons for continuing use	Produces state of inner peace [1]	Started at small dose and worked up [1], to compare the effects [1]
Method of consumption	Seeds eaten [2], seeds consumed in water [1], fresh leaves as tea [1], fresh leaf eaten raw [1], usually seeds but sometimes leaves or roots made into tea [1], *D*. *innoxia* leaves as tea [1]	Ate seeds [3], infused seeds in water [1], infused ground seeds in water before allowing to evaporate and then infusing in alcohol [1]
Dose	Small dose 10 seeds and large dose 70 seeds [1], two big handfuls of leaves in a teapot and drank 200–300 ml [1], 1–10 seeds [1], 2 seeds [1], ate a thumb-sized piece of leaf [1]	5–25 seeds [2], average listed on Erowid [1], 150 seeds [1]
Use with other substances	Alone [2], with alcohol [2]	Alone [2], with alcohol a couple times [1], with garlic [1]
Time of use	Evening [2], weekend [1], summer [1]	Day [2], evening [1], morning [1], weekend [1], spring [1], summer [1]
Place of use	Outside [3], inside and outside [1], at home [1]	Home [3], inside and outside [1]
Use with others or alone	With others [3], alone [1]	Alone [2], alone or with others [2]
Methods of obtaining	Collected in nature [3], grows the plant at home [1]	Garden store [2], collected from a home garden [2]
State when obtained	Fresh [5]	Seeds [4]
Cost	Free [5]	Not sold [2], 1€ for 30 seeds [1], 2€ per package of seeds [1]
Effects	Complete loss of touch with reality [2], distortion of time [2], vivid dreams with demonic symbols and worst fears [1], no memory of events while high [1], walked naked through city [1], speaking to self [1], waking up 40 km away from home [1], fatigue [1], seeing/talking to people who did not exist [1], realistic hallucinations lasting 24 hours after ingestion [1], seeing stars floating around [1], feeling of face turning into a cat's [1], fogginess of vision [1], distortion of space [1]	Nausea [2], vivid imagination [2], no effects [2], dizziness [1], hyper attention to sound [1], closed eye visuals [1], feeling of skin moving [1], similar to LSD [1], fatigue [1], vivid colours [1], feeling sound [1], childlike fantasies [1], tactile enhancement [1], ego dissolution [1], disconnection from self [1]
Dangers	Poisonous [4]	Chemically treated seeds [1], getting hurt while high [1], dangerous [1], not dangerous [1], derealisation [1], panic attacks [1]
Natural knowledge	White flowers [2], Solanaceae [1], came to Europe from the Americas [1], white trumpet flowers in summer [1], dark green bush [1], grows in Slovenia [1], grows in wild and degraded soils [1], abundant in coastal region of Slovenia [1]	Light green heart-shaped leaves [4], vine [3], sweet potato relative [1], trumpet flowers in many colours [1], common garden plant [1], small dark wedge seeds [1], blue flowers [1], flowers that last one day [1], seeds in pods [1], from South America [1], from North America [1]

### *Ipomoea* spp.

Members of the Convolvulaceae family, *Ipomoea* L. spp. are vines from the Americas that are now widespread garden plants [14, 56]. The taxonomy is complicated as both *I*. *tricolor* Cav. and *I*. *violacea* L. are considered hallucinogenic, though many authorities say they are the same species; similarly, *I*. *purpurea* (L.) Roth is sometimes stated to be hallucinogenic, though many believe it is not [14, 57]. The seeds of hallucinogenic species contain ergine, otherwise known as lysergic acid amine (LSA), a substance similar to LSD [14, 16, 56]. Research in Poland suggests usage for these plants as hallucinogens is low, stating that the unpleasant gastrointestinal side effects may deter users [57]. Toxic coatings used on seeds sold in stores may likewise be a deterrent [16]. A study in France found less than one third of hallucinogenic plant users had employed these seeds [8].

In the present study, 5 individuals listed this plant, most having heard about it through the internet. Curiosity was the main reason to try it. Those who used it multiple times did so as they felt they had begun at too low a dose. Unpleasant side effects were the reason for discontinuing.

All participants used the seeds: 3 eating them, one infusing them into water, and one infusing ground seeds into water, which was then evaporated and infused in alcohol. Most tried 5 to 25 seeds, but one used 150. One participant took garlic with the seeds, believing this would combat the nausea. All participants used this plant at home, and almost all while alone.

2 bought the seeds from garden stores, selecting those without toxic coatings. 2 collected the seeds from gardens of people they knew. The price was suggested to be 1–2€ per package (~30 seeds), while those who collected the seeds had never heard of them being sold.

The most frequently reported effects were nausea, heightened imagination, and no effect. One individual began experiencing panic attacks regularly after using these seeds, a state that lasted for a year and even caused agoraphobia.

Responses are summarised in Table 6.

### Lophophora williamsii

A small, slow-growing cactus found around the border of the USA and Mexico, this member of the Cactaceae family is known for its ritual use that may extend back almost 6000 years [14, 16, 56]. The hallucinogenic effects of this cactus are attributable to mescaline, though some theorise that other compounds also play a role [56, 58]. Research in France found 9 of 30 participants had used either *L*. *williamsii* (Lem. ex Salm-Dyck) J. M. Coult or *E*. *pachanoi*, primarily by making them into tea, cooking them and creating a dough from them, or merely swallowing them raw [8].

5 participants in the present study listed this plant, mainly first having heard of it through books and trying it due to curiosity. Most used this cactus only once, with availability keeping them from further experiences, though one individual stated that they ceased using it out of respect for the plant. Buttons were eaten raw or dried, ground into water, or made into tea. One participant used marijuana later in the trip to enhance the effects. It was primarily used in nature.

Most used the plant with other people, one during shaman-led ceremonies. Two participants grow the plant themselves after finding it at local flower shops where it was being sold as a house plant. Participants mentioned a range of mild hallucinogenic effects. Antidepressant aftereffects were also reported.

Responses are summarised in Table 7.

**Table 7:** Overview of *Lophophora williamsii* and *Tabernanthe iboga*.

	*Lophophora williamsii*	*Tabernanthe iboga*
How first heard of	Books [3], friends [1], internet [1]	Books [2], internet [1]
Why first tried	Curiosity [3], offered by a friend [1], experience another reality and reduce ego [1]	Curiosity [2], diminish ego [1]
Number of times used	Once [3], 3 times [1], twice per year [1]	2 large doses when younger and now microdoses twice per year [1], once [1]
Reasons for discontinuing use	Availability [2], respect for plant [1]	Stopped with large doses because no longer needed to learn from the plant [1], only available once [1]
Reasons for continuing use	Experience power of the plant [1]	Microdoses to strengthen/clean/sharpen mind [1]
Method of consumption	Chew raw or dried buttons [2], tea [1], dried buttons ground into water [1]	Powdered root bark or alcohol extraction eaten [1], cream on skin [1]
Dose	12 small buttons [1], about 1.5 g [1], unsure [1]	Microdoses: 5 g of bark or 50 mg of extract [1]
		Full doses: 30 g of root bark or 1.2 g of extract [1]
Use with other substances	Alone [3], with marijuana [1]	Alone [1]
Time of use	Varies [2], morning [1]	Varies [1]
Place of use	Nature [3], inside [1]	Home [1], inside with shaman [1], at conference [1]
Use with others or alone	With others [2], alone and with others [1], alone [1]	Alone [1], with shaman [1]
Methods of obtaining	Bought in local flower shop [2], from friend [2], from shaman [1]	Brought back from Gabon [1], from shaman [1], at conference [1]
State when obtained	Fresh [3], dried [1], beverage [1]	Powdered root bark [1], shamanic preparation [1], cream [1]
Cost	10€ for a potted plant [1], 25€ for a potted plant [1], 100–250€ for a ritual incorporating it [1], unsure [1]	300€ per dose [1], 100–250€ for a ritual incorporating it [1]
Effects	Like *Psilocybe* spp. [2], contextual effects [1], altered reality [1], teaches lessons [1], like LSD [1], bright colours [1], vivid imagination and daydreaming [1], altered colours [1], sense of connection to whole world [1], antidepressant aftereffects [1]	Enlightening [1], like ayahuasca [1]
Dangers	Psychologically dangerous [2], bad trip [1]	Hard on the heart [1], dangerous when combined with drugs [1]
Natural knowledge	From South America [1], endangered [1], from desert of North America [1], small cactus with little purple flowers [1]	Yellow fruits [1], from the jungle in Africa [1]

### Tabernanthe iboga

Africa is the place of origin of few hallucinogenic plants, but the most famous is *Tabernanthe iboga* Baill. [59]. From the alkaloid-rich Apocynaceae family, this plant is known for its ritual use among the Bwiti [47, 56]. The hallucinogenic alkaloid, ibogaine, inhibits serotonin transport in the central nervous system to cause its psychoactive effects [60]. It seems to not have found much use outside of Africa; a study in France found only one participant of 30 had used this plant, doing so after getting it as a powder and rolling this into a ball to chew [8].

3 individuals listed this plant in the present study. One only used a small amount of cream with it at a conference, while the other 2 made greater use. The 2 repeat users were at odds as to the difficulty to obtain this plant: one participant brought it from Africa and listed it as 5 out of 5, while one participant receives it in ceremonies and listed it as 1 out of 5, saying that it is easy to get with the right contacts.

Responses are summarised in Table 7.

### Argyreia nervosa

From the family Convolvulaceae, this plant is from the Indian sub-continent, where it has traditionally been used for a range of medical conditions and is incorporated into the Ayurvedic pharmacopoeia [61]. It is now widely grown as an ornamental for its showy flowers, and the seeds are often coated with toxic substances as with *Ipomoea* spp. to discourage using them for their LSA content [16, 61]. First becoming widespread in the 1990s, research in France found that almost one third of study participants had tried this plant, often using 5–15 seeds [8]. This same study found that the seeds of *A*. *nervosa* (Burm. F.) Bojer were often used only once or twice, with low availability often cited as a reason for discontinuing [8]. A similar study in Poland noted that 6–8 seeds are normally employed, and states that gastrointestinal side effects were often what caused discontinuation, while, opposite to the previous study, stating that low price and ease of access were often reasons for use [57].

3 participants listed this plant in the present study, mainly hearing of it through the internet. One used the seeds just once due to accessibility and as they developed panic attacks after taking them, while one takes the seeds annually. Both ate the seeds without other substances, one taking 6–8 and the other unsure of the amount. Both took the seeds with other people; one added that the feeling of telepathy made taking them in a group best. One participant bought capsules filled with seeds in a shop, stating that they cost about 12€ for 4 capsules.

One participant likened the effect to *Ipomoea* spp. seeds, though more dangerous as it produces a more profound experience that was stated to affect women more than men, though the participant to claim this was a cisgender male.

Responses are summarised in Table 8.

**Table 8:** Overview of *Argyreia nervosa* and *Atropa belladonna*.

	*Argyreia nervosa*	*Atropa belladonna*
How first heard of	Internet [2], Blue light [1]	Internet [1], folk tradition [1]
Why first tried	Reach altered state of consciousness [1], curiosity and convenience [1]	Interest in effects [1], desire to join folk tradition [1]
Number of times used	Once [1], once per year [1]	A few times [1], twice per year [1]
Reasons for discontinuing use	Only had access once [1]	
Reasons for continuing use	Pleasant feeling of telepathy [1]	Enjoyed the effects [1], desire to feel the power of the plant [1]
Method of consumption	Seeds eaten [2]	Berries eaten or leaves cooked in wine [1], leaves cooked in coconut oil and applied to skin [1]
Dose	Unsure [1], 6–8 seeds [1]	Double handful of leaves in a handful of oil [1]
Use with other substances	Alone [2]	Alone [1]
Time of use	Day [1], evening [1]	
Place of use	Home [1], nature [1]	Nature [2]
Use with others or alone	With others [2]	Alone and with others [1], with others [1]
Methods of obtaining	From a friend [2], from a store [1]	Collected in nature [2]
State when obtained	Seeds [2], seeds in capsules [1]	Fresh [2]
Cost	Unsure [2], 12€ for 4 capsules [1]	Free [2]
Effects	Telepathy [1], clarity to life problems [1], similar to *Ipomoea* spp. [1], vivid colour vision [1], enhanced imagination [1], feeling sounds [1], nausea [1], anxiety [1]	Unable to describe [1], seeing dark things better than brighter things [1], feeling of lightness in legs [1]
Dangers	Can cause vasoconstriction leading to loss of extremities and organ damage [1], many people have bad trips on it [1], more dangerous than *Ipomoea* spp. [1]	Dangerous if used foolishly [1], as dangerous as other psychoactive substances [1]
Natural knowledge	Large colourful flowers [3], from South America [1], looks like *Datura* spp. [1], from the Americas [1], from Hawaii [1], grows as a bush [1]	Grows in Slovenia [2], grows in higher altitude where trees have been cut [1], beautiful berries [1]

### Atropa belladonna

Native through parts of Europe, this member of the Solanaceae was the first plant to have the tropane alkaloid atropine (the racemic mixture of hyoscyamine enantiomers) isolated from it [36, 51, 62]. Its hallucinatory and lethal effects have been known since ancient times, and many claim that it was once used in the ointments that witches of Medieval Europe would use to give themselves the illusion of flying [56, 62]. It was heavily abused during the mid-to-late 1900s as it was used in commercial products for asthma; these products were often used as recreational drugs, frequently with dangerous results [63]. Even since the discontinuation of such products, *Atropa belladonna* L. has found use as a hallucinogen. A study of Spanish poison control found that 7.3% of the calls related to intentional plant exposures were due to this plant, while a German study of inpatients with substance abuse problems displayed a fair number of users [10, 54]. A similar study carried out in Slovenia displayed that *A*. *belladonna* was the fourth most common cause of plant poisonings in the country, with these cases being a mix of attempted suicides and recreational use [11]. Nearby in the Bilogora region of Croatia, there even existed a tradition of boys taking the berries to amuse themselves during time spent herding flocks in the mountains [64].

In the present study, *Atropa belladonna* was listed by 3 participants. One read about the plant on the internet, and used it a few times since they liked the effect; another learned of it through existing folk traditions and as part of this uses it about twice per year. The regular user does so by eating the berries or cooking the leaves in wine, while the participant to use the plant a few times did so by cooking dried leaves in coconut oil to make an ointment, usually applied in nature. They noted lightness of their legs and changes to their visual perception, with it being easier to see darker rather than lighter areas; these symptoms would make sense, given that the alkaloids of this plant are said to have caused a feeling of flying when used by European witches and since they dilate the pupils, which would allow more light into the eyes [16, 56]. This plant was used alone, though the regular user noted that they occasionally added the leaves to *Hyoscyamus niger* L. ointments. Both collect the plant in nature.

Responses are summarised in Table 8.

### *Artemisia* spp.

A member of the Asteraceae family, *Artemisia* L. spp. are famous for the role of *A*. *absinthium* L. in making absinthe. Though there are many debates around whether or not absinthe was hallucinogenic, many *Artemisia* spp. produce a range of secondary metabolites, the most known perhaps being thujone, a GABA receptor modulator [65, 66]. Though not proven hallucinogenic, many believe it to be, and some have gone so far as to try it themselves to confirm this [67].

3 participants listed *Artemisia* spp. in the present study. Only one used it regularly, doing so over 5 years in the form of alcoholic beverages. It was curiosity that drove 2 participants to try *Artemisia* spp., while the third did so to increase the intensity of their dreams. No hallucinogenic effects were reported. The individual who consumed the plant in alcoholic beverages believed it is not dangerous in alcohol, saying that this cancels out the GABA inhibiting effect that they ascribe to the plant, but that otherwise it is harmful.

*Artemisia* spp. were the only plant or mushroom listed by one of the participants, something that was otherwise seen only for ayahuasca and *Psilocybe* spp. It stands out from the other two, however, as being the only one that is traditionally used in Slovenia. *Pelinkovec*, an alcoholic beverage made by infusing *Artemisia* spp. into spirits, has an extensive history of use as both a medicine and recreational beverage in some parts of the country. It is thus unsurprising to see it listed alone, though odd that it was listed so few times in total; this may be tied in with perceptions of this plant and the lack of consensus as to its hallucinogenic nature, with many likely not mentioning it as they do not believe it to be psychoactive. This topic is further addressed in the limitations section at the end of this paper.

Responses are summarised in Table 9.

**Table 9:** Overview of *Artemisia* spp. and *Peganum harmala*.

	*Artemisia* spp.	*Peganum harmala*
How first heard of	Friends [1], commonly spoken of [1]	Book [1], friend [1], internet [1]
Why first tried	Curiosity [2], to increase dreaming [1]	Curiosity [2], enhance the effects of psilocybin [1]
Number of times used	Once [1], a few times [1], multiple times [1]	Once [2], 2 times [1]
Reasons for discontinuing use	Only experimenting [1]	Used other substances [1], didn't feel the need to repeat experience [1], hasn't had time to repeat experience [1]
Reasons for continuing use	Pleasant experience [1]	
Method of consumption	Alcoholic beverage of leaves [1], root tincture [1], tincture [1]	Seeds made into tea [1], cold water infusion of crushed seeds [1], eating the seeds [1]
Dose	Unsure [1]	5–10 g of seeds [1], 200–300 g of seeds [1]
Use with other substances	Tobacco [1], alone [1]	Alone [2], with *Psilocybe* spp. [1]
Time of use	Evening [1], night [1]	Day [1]
Place of use	Home [2], parties [1]	Home [2], cottage [1]
Use with others or alone	Other people and alone [1]	With others or alone [2], alone [1]
Methods of obtaining	From friends [1]	From a friend [2], from a shop [1]
State when obtained	Alcoholic beverage [1]	Seeds [3]
Cost	Varies [1]	Unsure [2], 10€ for 3–4 doses
Effects	No hallucinations [2], euphoria [1], increased imagination [1], increased energy [1], enhanced the effects of alcohol [1]	Similar to ayahuasca [1], unlike other hallucinogens [1], plastic quality to vision [1], antidepressant aftereffects [1], enhanced *Psilocybe* spp. trip [1]
Dangers	Not when used in alcohol [1]	Psychologically dangerous [1], dangerous since it is an MAOI [1]
Natural knowledge	Green spiky leaves [1], from central Europe [1], shrub [1]	Doesn't grow in Slovenia [2], small bush [2], from the Mediterranean [1], from the Middle East [1], white flowers [1], small leaves [1]

### Peganum harmala

From the family Nitrariaceae, this plant is from the Mediterranean region and Near East [68]. Its seeds are used medicinally for a range of ailments and the presence of the alkaloids harmine and harmaline also give it psychoactive effects [62, 68]. *P*. *harmala* L. has been known to cause a dream-like state at low doses and full-blown hallucinations at higher doses, while also being able to intensify the effects of other drugs [62, 68]. Its seeds are often seen as the best MAOI source outside of tropical regions and thus have been often used to make ayahuasca analogues, for example by combining it with *Phalaris* L. spp. [69, 70].

3 participants in the present study used this plant. Curiosity led 2 of the participants to try it, while the third used it to strengthen the effects of *Psilocybe* spp. This participant could not differentiate the effects of the plant from the mushrooms, though their trip started faster, hit harder, and lasted longer. The others described the effects as everything taking on a plastic-like quality. One participant stressed that as an MAOI this plant could be dangerous if combined with the wrong substances, possibly causing death; as such, they adjusted their diet to avoid foods like coffee, cheese, and chocolate before using it as they felt these would have a bad reaction with it.

Responses are summarised in Table 9.

### Others

Responses are for substances listed twice are summarised in Tables 10 and 11.

**Table 10:** Overview of *Scopolia carniolica* and *Mandragora* spp.

	*Scopolia carniolica*	*Mandragora* spp.
How first heard of	Folk tradition [1], urban legends [1]	Book [1], folk tradition [1]
Why first tried	Curiosity [1], desire to join folk tradition [1]	Curiosity [1], desire to join folk tradition [1]
Number of times used	Twice [1], twice per year [1]	Twice [1], multiple times [1]
Reasons for discontinuing use	Only experimenting [1]	Curiosity satisfied [1]
Reasons for continuing use	Underestimated dose first time [1]	
Method of consumption	Fresh rhizome grated into hot water [1], rhizome cooked [1]	Root cooked in fat and made into ointment [2], root cooked in wine and drank [1]
Dose	20 g of rhizome in 200 ml of water [1]	200 ml of wine infused with it [1]
Use with other substances	Alone [1]	Alone [2]
Time of use	Evening [1]	Variable [2]
Place of use	Inside [1], nature [1]	Dubrovnik [1], home [1], nature [1]
Use with others or alone	With others [2]	With others [1], alone [1]
Methods of obtaining	Collected in nature [2]	Collected with herbalist in Dubrovnik [1], collected in Slovenia [1]
State when obtained	Fresh [2]	Fresh [2]
Cost	Free [2]	Free [2]
Effects	Unable to describe [1], bad taste [1], difficulty walking [1], unable to sleep [1], itchy skin [1], hot/cold flashes [1], realistic hallucinations [1], dilated pupils [1], difficulty seeing close up and reading [1],	Unable to describe [1], increased strength [1], desire to fight [1], aphrodisiac [1]
Dangers	Dangerous [1], dangerous if used foolishly [1]	Dangerous if used foolishly [1], atropine and scopolamine dangerous in large doses [1]
Natural knowledge	Pointed green leaves [2], purple flowers [1], among first plants to grow in spring [1], ginger-like rhizome [1], grows in Slovenia [1]	Purple flowers [2], low and leafy [2], dormant most of year underground [2], grows in Slovenia [1], does not grow in Slovenia [1]

**Table 11:** Overview of *Mimosa hostilis* and changa.

	*Mimosa hostilis*	Changa
How first heard of	Magazine [1], friend [1]	Internet [1]
Why first tried	Curiosity [1], availability [1]	Availability [1]
Number of times used	Once [1], weekly and then down to every couple of years [1]	5–6 times [1]
Reasons for discontinuing use	Availability [2]	
Reasons for continuing use	Enjoyable experience [1]	Strong positive effects [1]
Method of consumption	Root bark extract smoked [2]	Smoking [1]
Dose	Unsure [1], 50 mg of extract [1]	0.1–0.2 g [1]
Use with other substances	Alone [1], sometimes with alcohol or MDMA [1]	Alone [1]
Time of use	Varies [1]	Varies [1]
Place of use	Inside [2]	Home usually [1]
Use with others or alone	With others [2]	Usually with others [1]
Methods of obtaining	Bought from the internet [1], from friend [1]	Brother [1], dealer at music festival [1]
State when obtained	Root bark powder [1], extract [1]	Mix of finely chopped plant material [1]
Cost	100–200€ for 1 kg of root bark [1], unsure [1]	100€ / g [1]
Effects	Like stepping into another universe [1], like being shot into a mandala [1], contact with entities [1], experienced death [1], nausea [1], anxiety [1], intense audio and visual distortions [1], gives answers to life problems [1]	Not like *Psilocybe* spp. [1], fast onset [1], strong patterned visuals [1], everything seeming to come to life [1], everything seeming to breathe and move [1], vivid colours [1], feeling of calmness [1], emotional stability [1], extensive aftereffects lasting for days [1]
Dangers	If you take too much [1], if you are in a bad location and panic [1]	Dangerous for those with mental health issues [1]
Natural knowledge	Unsure [1], from South America [1]	*Peganum harmala* and *Passiflora* spp. mixed in [1]

Responses for substances listed only once are summarised in Table 12.

**Table 12:** Overview of other substances.

	Age of first use	Age of last use	How learned of	Reason to try	Frequency of use	Reason for number of uses	Parts used and preparation	Amount	Use with other substances	Time of use	Place of use	Use alone/in company	Method of obtaining	Difficulty to obtain (1 easy to 5 hard)	Form obtained in	Cost	Experience	Dangers	Natural knowledge
***Psychotria viridis***	50+	60	Books, personal contacts, internet	Experience other reality, curiosity, diminish ego			Smoked						Shaman	1–2, easy with connections	Prepared	100–250 euros for rituals	Visual and auditory effects, fractals, classic travels in universe and earth, related to nature.		
***Spartium junceum***	19	19	Internet	Lucid dreaming	Once	Availability	Seeds dried and eaten	9 seeds	Alone	Before sleep	Home	Alone	Nature	1	Dried seeds	N/A	Meditation-like feeling, dreams with intense colours	Overdose	Shrub, grows along coast, yellow flowers, Fabaceae, pod for its fruit.
***Myristica fragrans***	16	16	Internet and friends	Substitute for marijuana	Once	Minimal effects, multi-day "hangover"	Spice mixed with juice	2–5 g	Alone	Day	Home	Alone	Kitchen	1	Powdered		Difficult to notice	Very addictive	
***Hyoscyamus niger***	22	24	Folk tradition	Folk tradition	~twice per year	The desire for the power of the plant	Ointment of leaves with belladonna leaves.			Depends	Nature	With others	Nature	1	Fresh	N/A	Indescribable	If used carelessly	Grows in Slovenia though rare
**LSA (powder in a capsule)**	24	24	Friends and internet	New perspective, therapeutic benefits	Once	Nausea	Powder in capsule	1 capsule	Alone	Day	Home	With others	Head shop	1	Powder in capsule		Increased detail to vision and thoughts, mild visual distortions, relaxing of body, increased empathy, nausea, sense of purpose to world	Bad environment, wrong dose sizes	Found all over Slovenia, mould or fungus that grows on grains
***Delosperma acuminatum***	37	37	Internet	Curiosity	Once	Impractical preparation	Stems and leaves, extraction	0.2 g	Alone		Home	Alone	Nature	3, grows in few locations	Fresh	N/A	Travel to another dimension, strong visual/auditory distortion	Psychological dangers	Not found in Slovenia, but on the islands off Croatia
***Phalaris arundinacea***	38	38	Internet	Testing plants	Once	Impractical preparation	Stems and leaves, extraction	0.2 g	Alone		Home	Alone.	Nature	3, difficult to find	Fresh.	N/A	No effects	Psychological dangers	Difficult to find in Slovenia
***Laburnum anagyroides***	25	25		Sanding wood	Once	Accidental	Wood dust										Whole body sense of jitteriness		Grows in specific altitudes normally on steep slopes, likes sun.
***Laetiporus sulphureus***	28	28	Friend.	Hunger	Once	Nausea	Young parts baked and eaten	Three chunks	Alone		Home	Alone	Nature	3	Fresh	N/A	Nausea, followed by changes in perception akin to *Psilocybe* spp.	Sensory issues	Yellow tree mushroom found in Slovenia

### Limitations

As recruitment was largely carried out through word of mouth, many participants knew each other. As such, this is not a random sample. Combined with the use of social media, this may have led towards a younger demographic being studied.

As with any study based on self-report, the present study is limited to assuming responses were truthful. As drugs may be a taboo topic, it may be difficult to obtain honest answers. However, individuals uncomfortable answering such questions would likely not participate. Participants were, in fact, quite eager to share their experiences and knowledge about this topic, and expressed excitement that the subject was garnering academic attention.

All plants and mushrooms that participants listed have been incorporated here even if not proven hallucinogenic, as the belief that a substance is hallucinogenic is more important in an anthropological context than its objective effects. However, participants were told in advance that marijuana use was not a focus of the study since marijuana is not traditionally grouped with hallucinogens and is only hallucinogenic in extreme doses. Additionally, its popularity would likely have skewed this study away from its focus.

Identification issues may also be present, though the participants were likely knowledgeable enough to know what they were getting and using, especially as it usually came from trusted sources.

## Conclusion

In academia, non-problematic drug use is often ignored, with dangerous and problematic cases often being used as an archetype for all substance use; indeed, when studies focus on this aspect, they are sure to produce biased results [71]. In the present study of 68 users of hallucinogenic plants and mushrooms in Slovenia, a great deal of non-problematic drug use has been recorded. Furthermore, much of this use was even described as being beneficial in nature. Rather than seeing all drug use as problematic, it is time for a more modern and science-based approach to replace the engrained religious-moral view that assumes all altered states of mind (or the substances that cause them) are "bad" and harmful.
